# Anti-Platelet Therapy with Cangrelor in Cardiogenic Shock Patients: A Systematic Review and Single-Arm Meta-Analysis

**DOI:** 10.3390/medicina60122092

**Published:** 2024-12-21

**Authors:** Jacopo D’Andria Ursoleo, Luca Baldetti, Marina Pieri, Pasquale Nardelli, Savino Altizio, Silvia Ajello, Anna Mara Scandroglio

**Affiliations:** 1 Department of Anesthesia and Intensive Care, IRCCS San Raffaele Scientific Institute, 20132 Milan, Italy; dandria.jacopo@hsr.it (J.D.U.); nardelli.pasquale@hsr.it (P.N.); altizio.savino@hsr.it (S.A.); scandroglio.mara@hsr.it (A.M.S.); 2 Cardiac Intensive Care Unit, IRCCS San Raffaele Scientific Institute, 20132 Milan, Italy; luca.baldetti@gmail.com (L.B.); ajello.silvia@hsr.it (S.A.); 3 School of Medicine, Vita-Salute San Raffaele University, 20132 Milan, Italy

**Keywords:** acute myocardial infarction, cangrelor, cardiogenic shock, mechanical circulatory support, percutaneous coronary intervention, VA-ECMO

## Abstract

*Background and Objectives:* Percutaneous coronary intervention (PCI) is a proven therapy for acute myocardial infarction (AMI) cardiogenic shock (CS). Dual anti-platelet therapy (i.e., aspirin plus an oral P2Y12 inhibitor) is recommended in patients treated with PCI. However, CS patients present severe hemodynamic instability, deranged hemostatic balance, and the need for invasive mechanical circulatory support (MCS) alongside invasive procedures, resulting in an increased risk of both bleeding and thrombotic complications, leaving uncertainty about the best anti-thrombotic treatment. Recently, the parenteral short-acting P2Y12 inhibitor has been increasingly used in the acute cardiac care setting, mainly in light of its favourable pharmacokinetic profile and organ-independent metabolism. *Materials and Methods:* In accordance with the Preferred Reporting Items for Systematic Reviews and Meta-Analyses (PRISMA) guidelines, we performed a systematic review and single-arm meta-analysis of the safety and efficacy outcomes (i.e., rates of major bleeding, occurrence of stent/any thrombosis, and hospital survival) of all existing original studies reporting on the intravenous administration of cangrelor in AMI-CS patients. *Results:* Ten studies (678 patients with CS) published between 2017 and 2023 were included in the present review: nine were observational and one had a randomized design. Percutaneous revascularization was performed in >80% of patients across the studies. Moreover, 26% of patients were treated with temporary MCS, and in all studies, concomitant systemic anticoagulation was performed. Cangrelor was administered intravenously at the dosage of 4 mcg/kg/min in 57% of patients, 0.75 mcg/kg/min in 37% of patients, and <0.75 mcg/kg/min in 6%. The pooled rate of major bleeding was 17% (11–23%, confidence interval [CI]), and the pooled rate of stent thrombosis and any thrombosis were 1% (0.3–2.3% CI) and 3% (0.4–7% CI), respectively. Pooled hospital survival was 66% (59–73% CI). *Conclusions:* Cangrelor administration in AMI-CS patients was feasible and safe with a low rate of thromboembolic complications. Haemorrhagic complications were more frequent than thrombotic events. Nevertheless, to date, the optimal dosage of cangrelor in this clinical context still remains not universally recognized.

## 1. Introduction

Cardiogenic shock (CS) is among the most severe presentations of acute heart failure (AHF) and is burdened by high morbidity and mortality [1]. Acute myocardial infarction (AMI) constitutes the most common cause of CS, accounting for more than 50% of cases [2,3]. Percutaneous coronary intervention (PCI) has dramatically reduced mortality rates in acute myocardial infarction–cardiogenic shock (AMI-CS) [4]. Therefore, AMI-CS patients will frequently have primary indications of dual antiplatelet therapy (DAPT). Nevertheless, these critically ill patients typically present a high risk of end-organ failure and deranged hemostatic homeostasis alongside a heightened risk of both bleeding and thrombotic complications. Furthermore, temporary mechanical circulatory support (tMCS) has been increasingly used to treat severe AMI-CS patients or cases complicated by cardiac arrest (CA) to allow hemodynamic support and/or resuscitation in the acute phase [5,6,7].

On one hand, while antithrombotic therapy holds a critical role in the minimization of stent- and plaque-related thrombotic complications (e.g., stent thrombosis and recurrent myocardial infarction), DAPT on top of continuous anticoagulation (triple antithrombotic therapy) required for tMCS carries relevant bleeding risk and holds the potential to negatively impact on patient outcomes [8,9].

Furthermore, the antiplatelet effects of oral P2Y12-receptor inhibitors exhibit a delayed and unpredictable effect in CA/CS patients due to slower and not reproducible absorption and metabolism of the medication coupled with difficulties in achieving sufficient enteral exposure in intubated patients [10,11,12].

Cangrelor, an intravenous non-thienopyridine, reversible adenosine-diphosphate P2Y12 receptor direct antagonist, has emerged as a potential adjunctive therapy due to its rapid onset and short duration of action [13].

By exhibiting a plasmatic metabolism, it is also particularly suitable for use in patients with liver and/or kidney dysfunction, both frequent scenarios in CS [14].

Being approved for administration in patients undergoing PCI who are P2Y12 antagonist-naïve and for whom therapy with oral P2Y12 antagonists is not feasible or desirable, its rapid onset and offset of action make this agent a particularly attractive option in patients at high risk of both thrombotic and hemorrhagic adverse events, hence mandating rapid titration of anti-thrombotic therapy [15,16].

Randomized data on cangrelor use showed improved major cardiovascular outcomes in AMI patients treated with cangrelor [17,18,19]; in addition, a single randomized controlled trial (RCT) confirmed the efficacy of a cangrelor maintenance dose of 0.75 mcg/kg/min in patients with acute coronary syndrome awaiting coronary artery bypass surgery [20]. Since the aforementioned dosage has been evaluated in hemodynamically stable AMI patients, these findings cannot be directly extrapolated to the CS setting, particularly due to potential safety concerns. The elevated risk of bleeding complications in AMI-CS patients, coupled with the critical need to establish an appropriate antithrombotic regimen, underscores the necessity for data specifically derived from this high-risk population. Notably, preliminary studies have begun to investigate the effects of a ‘low-intensity’ cangrelor regimen in patients receiving tMCS, thus offering valuable insights into this complex clinical scenario [21].

Although more experience is necessary to establish a unique recommended dose, preliminary evidence showed the safety and efficacy of reduced doses of cangrelor in patients at high bleeding risk, including doses lower than the Bridging Antiplatelet Therapy With Cangrelor in Patients Undergoing Cardiac Surgery (BRIDGE) trial dose (0.75 mcg/kg/min) [21,22].

**Figure 1:**
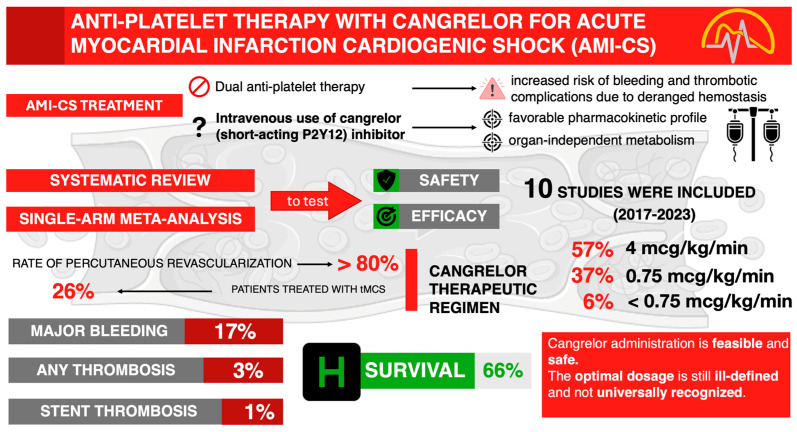
Visual abstract presenting main article structure, objective, research methodology, and results. AMI-CS: acute myocardial infarction–cardiogenic shock; tMCS: temporary mechanical circulatory support.

Considering this background, by synthesizing original data from the currently existing literature, this systematic review aims to provide insights into the role and clinical application of cangrelor in the high-risk population of patients undergoing emergent PCI in the context of AMI-CS, potentially guiding evidence-based clinical decision-making (Figure 1).

## 2. Materials and Methods

### 2.1. Search Strategy

**Figure 2:**
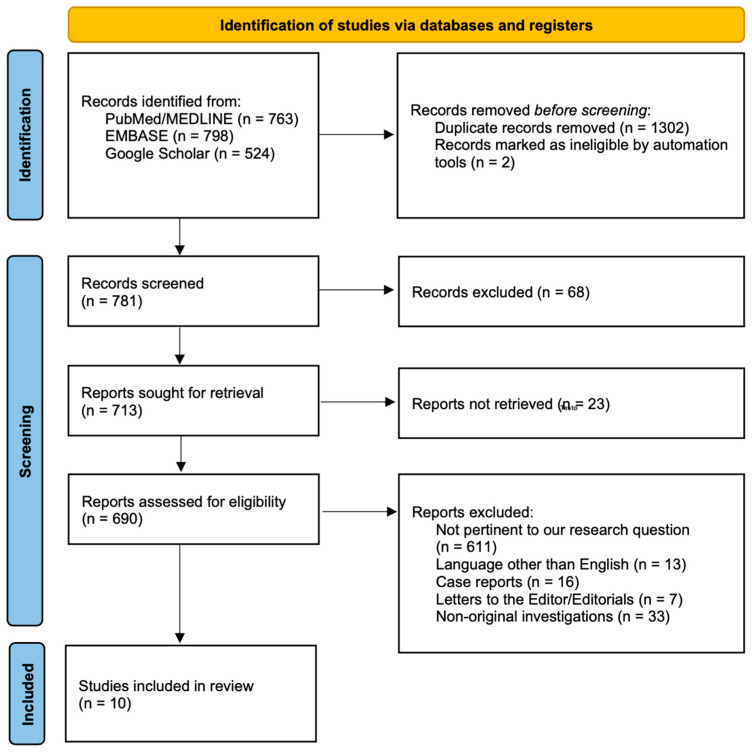
Flowchart of the studies selection and identification process.

As per the Preferred Reporting Items for Systematic Reviews and Meta-Analyses (PRISMA) checklist (Supplementary Material, Supplementary Table S1), two independent investigators (J.D.U. and L.B.) carried out a comprehensive search on PubMed/MEDLINE, EMBASE, and Google Scholar to identify relevant studies (up to 30 July 2024, without inception limits) (Figure 2).

A search strategy was planned to identify studies focused on “cangrelor” use in the context of “cardiogenic shock/cardiac arrest”, and the study protocol was registered on Open Science Framework with the unique registration DOI 10.17605/OSF.IO/AGTJ3. Due to the inconsistent usage of terms like CS across different studies, as well as due to the variety of tMCS available, synonymous terms and acronyms were incorporated in the search strategy, including “low cardiac output syndrome”, “percutaneous ventricular assistant device”, “intra-aortic balloon pump”, “Impella”, “extracorporeal membrane oxygenation (ECMO)”, “CS”, “AMICS”, “LCOS”, and “IABP” “PCCS”, “pVAD”, “Impella”, “ECMO”, “veno-arterial (VA)-ECMO”, and “counterpulsation”. Furthermore, the search strategy encompassed the inclusion of commercial names of cangrelor, namely “Kengreal” and “Kengrexal”, as well within the search string. Boolean operators (OR, AND) were utilized to combine keywords and free terms. Further information regarding the search strategy is made available in the Supplementary Material file (Supplementary Material, Search Strategy). Furthermore, the references of selected articles were scrutinized for potential additional studies to include (“snowballing”), which were also incorporated into this systematic review. Of note, only articles written in English were considered for potential inclusion.

### 2.2. Study Selection

Each reference obtained from the database search and literature search underwent independent evaluation by two investigators (J.D.U. and L.B.) at both title and abstract levels. In instances of concerns or disagreements, full-text articles were consulted, and disagreements were resolved by means of discussion.

#### 2.2.1. Inclusion Criteria

Studies and case series written in English reporting original experience of intravenous (iv) cangrelor administration in patients aged 18 years or older presenting with AMI-CS with or without the use of tMCS were identified and carefully assessed.

#### 2.2.2. Exclusion Criteria

Studies concerning the pediatric population, publications not presenting original data (including reviews, systematic reviews, meta-analyses, commentaries, conference abstracts that did not reach the full publication status, letters, and editorials), works published in languages other than English, and single case reports were excluded from this systematic review. No additional limitations on study design were applied.

### 2.3. Data Extraction and Study Characteristics

Data extraction was carried out employing the PICO (Patient/Population/Problem, Intervention, Comparison/Control, Outcome) approach and standardized forms. Specifically, the critically ill adult population presenting with AMI-CS with or without tMCS was considered the patient group. We assessed interventions involving cangrelor, alone or in combination with other anti-platelet drugs, versus any comparators when and if present, for managing anti-thrombotic therapy.

Abstracted information included details of the first author, publication year, and journal, patient characteristics (rationale for cangrelor administration and sample size), study design, type of stents deployed (if PCI was performed), type of tMCS, intervention details, presence of comparators, type of concomitant anticoagulation therapy (e.g., bivalirudin or unfractionated heparin and respective therapeutic regimen), treatment strategies (e.g., single or dual anti-platelet therapy and therapeutic regimen dose), relevant outcomes (e.g., 30-day, hospital, intensive care unit [ICU] and 1-year survival), clinical outcomes (e.g., major bleeding and thromboembolic events with the score used and the respective sites or locations).

The risk of bias assessment was performed with the Risk Of Bias In Non-randomised Studies—of Interventions (ROBINS-I) tool for observational studies and the risk-of-bias tool for randomized trials (RoB2) by Cochrane (accessible online at https://www.riskofbias.info, accessed on 10 August 2024), as shown in Supplementary Figure S1. Disagreements during the review process were resolved through discussions involving a third reviewer (M.P.) and by reaching a consensus.

### 2.4. Statistical Methods

Descriptive analysis was performed to report study characteristics and data. A single-arm meta-analysis was performed to assess outcomes of homogenous patients treated across different studies with regard to the antithrombotic regimen administered. In particular, the single-arm meta-analysis assessed the clinical outcomes of AMI-CS patients receiving systemic anticoagulation and DAPT (i.e., aspirin and cangrelor). Studies were included in the single-arm meta-analysis if they met all the following criteria: (i) cangrelor and systemic anticoagulation administered in all patients, (ii) aspirin administered in at least 70% of patients.

Pooled estimate rates and 95% confidence intervals (CI) of study outcomes were calculated using a random-effects model (DerSimonian and Laird). Analyses were conducted with Comprehensive Meta-Analysis v.2 (Biostat Inc., Englewood, NJ, USA). Higgins and Thompson’s I^2^ was employed to quantify the extent of statistical consistency [23]. An unadjusted *p*-value < 0.05 was considered statistically significant.

## 3. Results

### 3.1. Characteristics of the Studies

From the total number of 690 results assessed for eligibility, 10 investigations [9,21,24,25,26,27,28,29,30,31] were included for a total of 678 patients with AMI-CS.

All the studies were published in the 2017–2023 period: five studies (50%) were from the United States of America, and five studies (50%) were from European countries. A summary of the studies’ general details is reported in Table 1.

**Figure 3:**
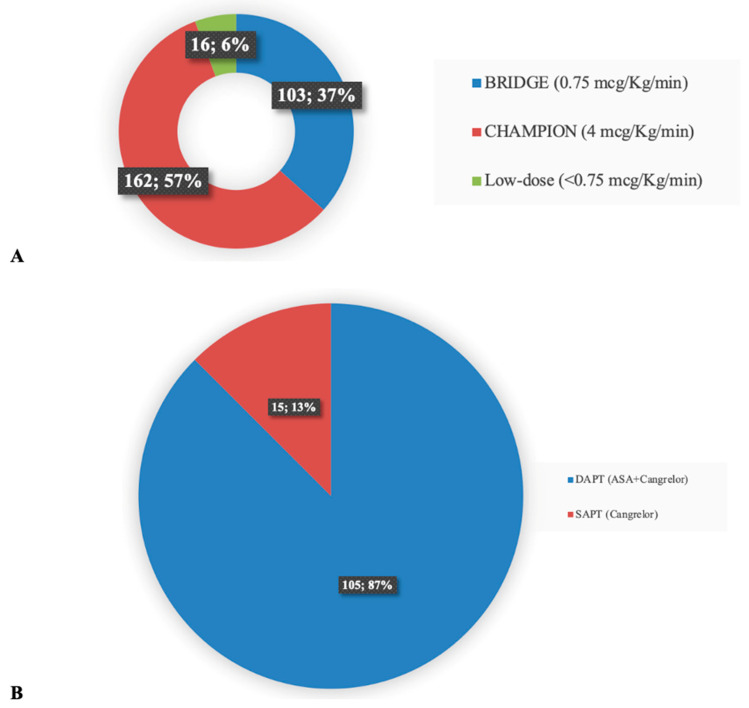
Dose of cangrelor in *n* = 281 patients from included studies (*n* = 10) (**A**). Therapeutic regimen of cangrelor in *n* = 120 patients from included studies (*n* = 10) (**B**). BRIDGE: The Bridging Antiplatelet Therapy with Cangrelor in Patients Undergoing Cardiac Surgery Trial; CHAMPION: Cangrelor Versus Standard Therapy to Achieve Optimal Management of Platelet Inhibition Trial; DAPT: dual antiplatelet therapy; ASA: aspirin; SAPT: single anti-platelet therapy.

The majority of them (9; 90%) [9,21,24,25,26,27,28,30] had a retrospective design, and one study was a prospective randomized trial sub-study [31]. Acute coronary syndrome was the cause of shock in all patients. All studies but one reported the PCI rate, which was >80% in all studies and 100% in six studies (60%). The cangrelor administration regimen and the related dose were dictated by clinical indication and are summarized in Figure 3.

**Figure 4:**
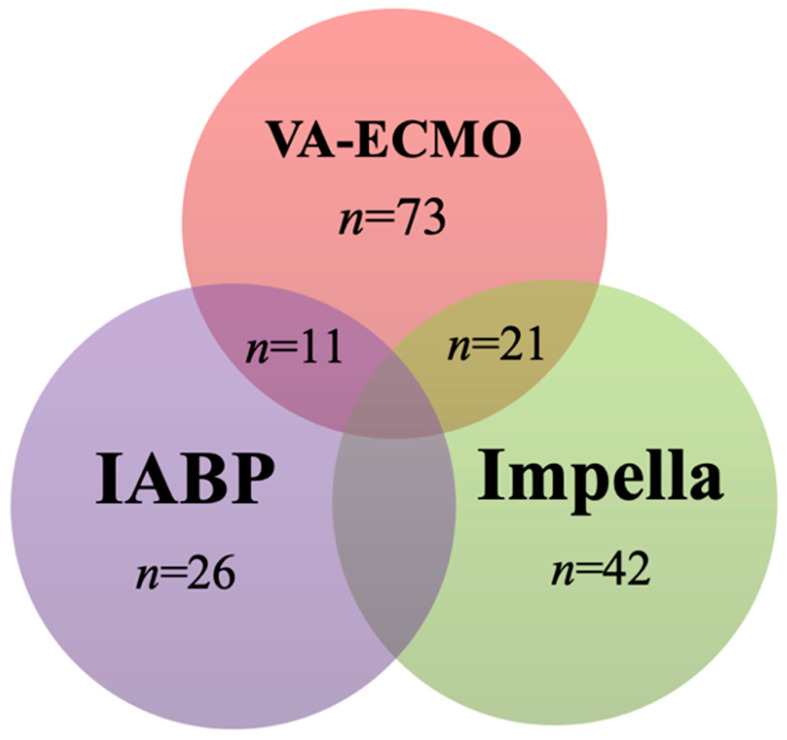
Type and strategy (single- or multi-device) of mechanical circulatory support (MCS) in *n* = 173 patients from included studies (*n* = 10). VA-ECMO: venoarterial extracorporeal membrane oxygenation; IABP: intra-aortic balloon pump.

Overall, the majority of patients (162, 57%) were administered a cangrelor dose of 4 mcg/kg/min, according to the indication of the Cangrelor Versus Standard Therapy to Achieve Optimal Management of Platelet Inhibition Trial (CHAMPION) trial [32]. A total of 103 patients (37%) received the BRIDGE trial dose of 0.75 mcg/kg/min [20], and 16 patients (6%) received a lower cangrelor dose (<0.75 mcg/kg/min) as determined by physician discretion, in view of their heightened risk of bleeding (Figure 3A). In most cases (105 patients, 87%), cangrelor was administered in the context of DAPT with aspirin, while in 15 patients (13%), cangrelor was administered as a single antiplatelet drug (Figure 3B). A vast number of patients with CS (173/678, 26%) received tMCS. The device types and their combinations are reported in Figure 4.

All the studies reported data on the occurrence of bleeding and thromboembolic complications. Further details about the occurrence of hemorrhagic and thromboembolic complications are reported in detail in Tables S2 and S3 in the Supplementary Material. Of note, the three studies reporting the higher rates of hemocompatibility complications, encompassing both thrombosis (14%, 13%, and 40%) and bleeding (49%, 77%, and 60%) [21,24,26], included only patients with temporary mechanical circulatory support.

### 3.2. The Effect of Cangrelor on the Rate of Major Bleeding, Thrombosis, and Hospital Survival

**Figure 5:**
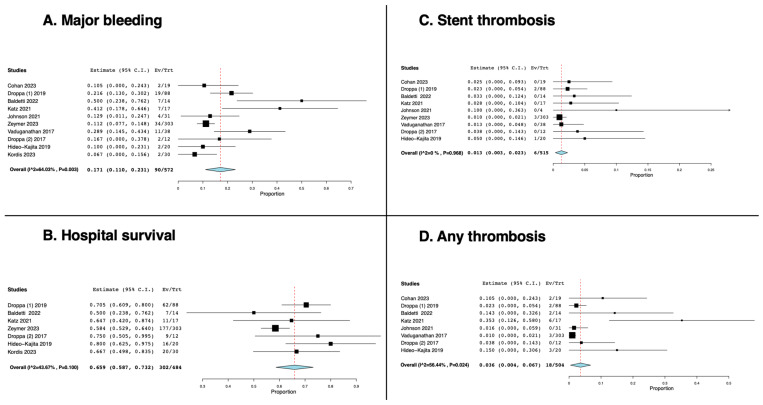
The effect of cangrelor on the rate of major bleeding (**A**) [9,21,24,25,26,27,28,29,30,31], hospital survival (**B**) [21,25,26,28,29,30,31], thrombosis (**C**) [9,21,24,25,26,27,28,29,30], and any thrombotic event (**D**) [9,21,24,25,26,27,29,30].

A single-arm meta-analysis was performed, including all the studies in which the same antithrombotic regimen (i.e., anticoagulation plus dual antithrombotic therapy) was administered to patients. As shown in Figure 5, the pooled rate of major bleeding was 17% (11–23% confidence interval [CI]), and the pooled rate of stent thrombosis and any thrombotic event was 1% (0.3–2.3% CI) and 3% (0.4–7% CI), respectively. Pooled hospital survival was 66% (59–73% CI) (Figure 5).

The pooled rate of the other reported bleeding and thromboembolic complications are reported in the Supplementary Material (Supplementary Figures S2–S7).

## 4. Discussion

We performed the first systematic review and meta-analysis on cangrelor use in AMI-CS patients. We found that cangrelor has been administered in the CS population at variable dosing regimens, chiefly according to the CHAMPION maintenance dose of 4 mcg/kg/min, followed by the BRIDGE maintenance dose of 0.75 mcg/kg/min. However, lower doses of cangrelor were also administered with neither efficacy nor safety issues, although the sample size of most studies was small. An antithrombotic regimen encompassing cangrelor was effective in preventing stent thrombosis in the AMI-CS population since such a complication was only rarely observed. On the contrary, the bleeding risk was higher than the thromboembolic risk in the critically ill population of CS patients, especially if tMCS is required, as further documented by the single-arm meta-analysis.

CS represents a heterogeneous clinical scenario with respect to the severity of patients’ clinical conditions, clinical evolution, and administered treatments. Since patients often exhibit hemodynamic instability and may require mechanical ventilation, the administration of parenteral instead of oral drugs such as cangrelor is supported by a strong rationale [10,11,33]. Among the various etiologies of CS, AMI presents the greatest challenges in terms of the management of antithrombotic therapy. On the one hand, PCI necessitates DAPT to prevent recurrent ischemia and ensure stent patency during the acute phase. On the other hand, AMI-CS patients exhibit unique characteristics, spanning thrombocytopenia secondary to CS or post-CA resuscitation, invasive procedural requirements, coagulation, inflammatory abnormalities, and end-organ dysfunction—all of which collectively heighten their bleeding risk. Additionally, when tMCS is required, full systemic anticoagulation becomes imperative, further complicating the antithrombotic strategy. Furthermore, shock-related end-organ function impairment may affect oral P2Y12 inhibitor metabolism, but cangrelor would not be affected by liver or kidney failure owing to its plasmatic metabolism [14]. Furthermore, the transition from cangrelor to oral P2Y12 inhibitors is straightforward, already routinely implemented in clinical practice, and can be initiated once the patient has achieved stabilization and has been weaned from tMCS based on clinical judgment. Despite the efficacy and safety profile of cangrelor in the acute cardiac care setting, the appropriate dose in patients with shock has never been directly addressed, with the majority of implemented protocols of therapeutic regimens deriving from previous studies on this drug (i.e., BRIDGE, CHAMPION). This aspect constitutes a knowledge gap that should be urgently addressed and supported by clinical data, given that our review also documented that bleeding events were more frequent than thromboembolic complications in AMI-CS. Moreover, in the absence of robust evidence, clinical management currently relies on decisions made by the Heart Team. This approach may contribute to an increased risk of complications and significant variability in treatment practices across different centers. As described in previous studies [34], antithrombotic management should be carefully titrated to suit CS clinical scenarios with antithrombotic regimens that prove to be individualized in terms of type of treatment, dose, and duration. In this setting, a platelet function test may confirm effective platelet inhibition at a given dosage [21]. Finally, the higher bleeding than thromboembolic rate may be partly ascribed to the presence of patients treated with MCS, which in turn is associated with non-negligible bleeding risk in the acute care scenario, which may also ultimately jeopardise patient outcomes [35].

Interestingly, hemocompatibility issues tend to occur more frequently in patients on extracorporeal support, and some thrombotic and hemorrhagic events—which are among the most frequently observed by the studies included in this review—are related to the presence of the MCS itself, such as groin/access site bleeding or thrombosis. This comes as no surprise, provided that the artificial surfaces of tMCS circuits serve as potent triggers for coagulation activation. Additionally, the components of these circuits impose mechanical shear stress and cause trauma to blood cells, while the duration of tMCS support—which can extend from days to several weeks—further exacerbates these effects. To further support this observation, the three studies reporting the higher rate of hemocompatibility complications, both thrombosis and bleeding, included patients with tMCS only [21,24,26]. Currently, a single-arm prospective study to investigate a less intense anti-thrombotic therapy featuring a bivalirudin plus an adjustable cangrelor dose in the setting of AMI-CS and VA-ECMO patients undergoing PCI is currently ongoing (SURVIVE, EudraCT number 2022-003199-17). Given this context, we advocate for further exploration of the most effective antithrombotic regimen required by AMI-CS patients. This entails documenting the optimal cangrelor dosage for this specific scenario, aiming to strike a balance between effectively preventing thrombosis and minimizing hemorrhagic complications. Ideally, such a study should differentiate between patients with and without MCS as distinct cohorts. Furthermore, as tMCS has become widely utilized over the past decades, and continuous technological advancements have led to the development of increasingly sophisticated devices for clinical application, this topic remains highly significant. Consequently, prospective data collection on the performance and outcomes of modern tMCS systems is critically needed.

### Study Limitations

This study suffers several limitations. First, all of the included studies were non-randomized in nature and featured heterogeneous populations, different indications for cangrelor use, and reported variable outcomes. Moreover, the small sample size of patients in the majority of the included studies may further hinder the generalizability of the findings. Second, no formal meta-analytic comparison between cangrelor-based and oral anti-platelet agent therapeutic regimens was run due to the limited number of studies that addressed this issue. Therefore, another limitation of our study lies in its descriptive design. Without a control group of patients treated with alternative oral P2Y12-receptor inhibitors, it is not possible to definitively evaluate the potential advantages or disadvantages of cangrelor therapy. Furthermore, anti-platelet therapy was often administered in conjunction with anti-coagulant therapy in very heterogeneous combinations across the included studies, and adherence to treatment was not reported to be consistent among studies, leaving uncertainty about which strategy may be preferable to the others. Analysing clinical practices implemented in the absence of consensus or formal guidelines—particularly given the lack of an approved cangrelor-specific dose for use in AMI-CS—is inherently constrained by significant heterogeneity in treatment approaches across different centers. Furthermore, despite extracting all available data, many critical details were not reported in the included studies. For instance, information on the discontinuation of DAPT or single anti-platelet therapy (SAPT) due to bleeding complications was absent, as were data on platelet count trends or results from platelet function assays. To conclude, due to the paucity of clinical data available, we were not able to perform separate analyses for patients with and without concomitant MCS. In the latter scenario, the presence of extracorporeal support is nevertheless known to exhibit a contribution to the occurrence of hemocompatibility adverse events.

## 5. Conclusions

Cangrelor was used in the high-risk AMI-CS population with variable dosing protocols. A relevant proportion of AMI-CS studies included patients supported by tMCS. The use of cangrelor in the context of triple anti-thrombotic therapy in AMI-CS patients resulted in a low rate of thrombotic events but substantial bleeding complications. Given the promising pharmacokinetic properties of this drug and the currently available body of evidence suggesting an excessive bleeding risk in the AMI-CS population, a solid background exists to prospectively test this agent in this setting, yet more data are needed to determine the appropriate posology in this specific patient population.

## Figures and Tables

**Table 1:** Summary of main characteristics of the included studies.

1st Author, Country	Journal, Year [Reference]	Sample Size	Study Design	Age, y	Male, Sex	Percutaneous Coronary Intervention Rate, %	Systemic Anticoagulation (Drug)	Concomitant Antithrombotic Drug(s) Administered with Cangrelor
Cohan, USA	Artif Organs, 2023 [24]	37	Retrospective	64 ± 11	62%	100%	heparin	aspirin
Droppa, Germany	Resuscitation, 2019 [25]	176	Propensity matching from trial	71 (59–77)	72%	93%	heparin or bivalirudin	aspirin
Baldetti, Italy	ASAIO J., 2022 [21]	14	Retrospective	58 (54–67)	93%	86%	bivalirudin	none
Katz, USA	Ann Pharmacother., 2021 [26]	17	Retrospective	65 (54–71)	82%	100%	heparin	aspirin
Johnson, USA	J Invasive Cardiol., 2021 [27]	31	Retrospective	62 ± 10	74%	not specified	heparin	not specified
Zeymer, Germany	Eur Heart J Acute Cardiovasc Care, 2023 [28]	303	Retrospective	65 ± 13	78%	100%	not specified	not specified
Vaduganathan, USA	JACC Cardiovasc Interv., 2017 [29]	38	Retrospective	65 (56–76)	63%	82%	heparin or bivalirudin	aspirin
Droppa, Germany	Cell Physiol Biochem., 2017 [30]	12	Retrospective	65 ± 3	83%	100%	bivalirudin	aspirin
Hideo-Kajita, USA	Am J Cardiol., 2019 [31]	223	Retrospective	63 ± 11	65%	100%	heparin or bivalirudin	aspirin
Kordis, Slovenia	EuroIntervention, 2023 [32]	91	Randomized controlled trial	not specified	not specified	100%	heparin	aspirin

## Data Availability

Further information is available from the corresponding author upon reasonable request.

## Supplementary Materials

The following supporting information can be downloaded at: https://www.mdpi.com/article/10.3390/medicina60122092/s1, Search string; Table S1: PRISMA checklist; Table S2: Incidence and site of bleeding; Table S3: Incidence and site of thromboembolic complications; Figure S1: Risk Of Bias In Non-randomised Studies—of Interventions (ROBINS-I) evaluation of included observational non-randomised studies (A). Risk-of-bias tool for randomized trials (RoB2) evaluation of included randomized trials (B); Figure S2: Pooled estimate of gastrointestinal bleeding; Figure S3: Pooled estimate of groin bleeding/cannulation site bleeding; Figure S4: Pooled estimate of intracranial hemorrhage; Figure S5: Pooled estimate of bleeding in the respiratory tract; Figure S6: Pooled estimate of retroperitoneal bleeding; Figure S7: Pooled estimate of ischemic stroke.
